# The Microbiome and Preterm Birth: A Change in Paradigm with Profound Implications for Pathophysiologic Concepts and Novel Therapeutic Strategies

**DOI:** 10.1155/2018/7218187

**Published:** 2018-10-02

**Authors:** Birte Staude, Frank Oehmke, Tina Lauer, Judith Behnke, Wolfgang Göpel, Michael Schloter, Holger Schulz, Susanne Krauss-Etschmann, Harald Ehrhardt

**Affiliations:** ^1^Department of General Pediatrics and Neonatology, Justus-Liebig-University and Universities of Giessen and Marburg Lung Center (UGMLC), Member of the German Lung Research Center (DZL), Feulgenstrasse 12, D-35392 Gießen, Germany; ^2^Department of Gynecology and Obstetrics, Justus-Liebig-University, Feulgenstrasse 12, D-35392 Gießen, Germany; ^3^Department of General Pediatrics, University Clinic of Schleswig-Holstein, Campus Lübeck, Lübeck, Germany; ^4^Research Unit for Comparative Microbiome Analysis, Helmholtz Zentrum München GmbH, Ingolstädter Landstr. 1, D-85764 Neuherberg, Germany; ^5^Institute of Epidemiology, Helmholtz Zentrum München, German Research Center for Environmental Health, Ingolstädter Landstr. 1, D-85764 Neuherberg, Germany; ^6^Comprehensive Pneumology Center Munich (CPC-M), Member of the German Center for Lung Research (DZL), Max-Lebsche-Platz 31, D-81377 Munich, Germany; ^7^Research Center Borstel, Leibniz-Center for Medicine and Biosciences, Borstel, Germany, Member of the German Center for Lung Research (DZL), Germany; ^8^Institute of Experimental Medicine, Christian-Albrechts-University of Kiel, Kiel, Germany

## Abstract

Preterm birth poses a global challenge with a continuously increasing disease burden during the last decades. Advances in understanding the etiopathogenesis did not lead to a reduction of prematurely born infants so far. A balanced development of the host microbiome in early life is key for the maturation of the immune system and many other physiological functions. With the tremendous progress in new diagnostic possibilities, the contribution of microbiota changes to preterm birth and the acute and long-term sequelae of prematurity have come into the research focus. This review summarizes the latest advances in the understanding of microbiomes in the amniotic cavity and the female lower genital tract and how changes in microbiota structures contribute to preterm delivery. The exhibition of these highly vulnerable infants to the hostile environment in the neonatal intensive care unit necessarily entails the rapid colonization with a nonbalanced microbiome in a situation where the organism is still very prone and at an early stage of development. The global research efforts to decipher pathologic changes will pave the way to new pre- and postnatal therapeutic concepts.

## 1. Introduction

Microbiomes comprise commensal, symbiotic, and pathogenic bacteria, fungi, and viruses, which form an ecological entity and interact with themselves and with their particular host. For a long time, it has been assumed that microbiota colonization is restricted to body surfaces like skin and the gastrointestinal tract. However, it became clear in the recent years that microorganisms reside in nearly every human tissue including the mammary glands, the ovaries, the uterus, and the placenta. Thus the human body is colonized by trillions of microbial inhabitants. They constitute a diverse and individually varying ecological community which in addition changes with age [1]. In line with this, the former theory that the amniotic cavity constitutes a sterile environment had to be abandoned. It became clear that the healthy maternofetal unit is colonized with microbes and that this is a prerequisite for immune maturation as well as metabolic and hormonal homeostasis. Fetal life and the first year of life are a life span which is critical for the development of a well-functioning immune system and maintenance of long-term health. For these reasons, negative early-life events pose a special risk to somatic and psychomotor development. Pathologic changes in microbiomes predispose or contribute to acute and chronic morbidities of every organ at any age. Furthermore, functional changes in microbiomes trigger infectious complications. In this review we present the latest insights into the structure and function of our microbiome and how pathologic changes contribute to preterm labor, premature birth, and the acute and long-term sequelae of prematurely born infants. Despite the fact that fungi and viruses are part of microbiomes, most research efforts so far clearly focused on bacterial diversity. Thus, other microbes than bacteria are mostly excluded in this review, simply because no data is available.

## 2. Functional Traits of the Microbiome of the Lower Urogenital Tract and the Fetomaternal Unit

The amniotic cavity has long been viewed as a sterile environment where the fetus is protected from the harmful external influences and threats. First reports emerged with the beginning of the new millennium, which questioned this hypothesis and described an intrauterine and placental microbial environment [2–4]. With the advancements in molecular techniques it became clear that the placenta, the amnion, and the fetus share large proportions of a common microbiome and that the maternal microbiome drives the development of the fetal immune system [5, 6]. The placental microbiome, under physiologic conditions, harbors nonpathogenic commensals including Firmicutes, Tenericutes, Proteobacteria, Prevotella, Neisseria, Bacteroidetes, and Fusobacteria but also potential pathogenic species like* Escherichia coli* [7]. As its detection is based on molecular techniques, the scientific discussion is ongoing whether the placental microbiome contains viable microbiota or just microbial components. Nonetheless, a regulatory function is currently assumed [8].

The vaginal microbiome resembles that of the cervix and is physiologically dominated by Lactobacillales, but Clostridiales, Bacteroidales, and Actinomycetales are also regularly detected. Bacterial community differences and shifts are almost exclusively detected between different* Lactobacillus* strains without any negative impact on pregnancy outcomes. During pregnancy, microbial richness and diversity are reduced along with an increased bacterial load. At the same time, the prevalence of potential pathogens like microbial species of ureaplasma and mycoplasma is reduced. The taxonomic composition of the microbial community of the vagina remains stable during pregnancy with an increase of the microbial diversity before birth of the healthy infant at term. These data lead to the conclusion that the composition of the vaginal microbiota is tightly regulated during pregnancy and that the switch to the nonpregnant situation precedes and maybe even triggers birth [9–13].

Microbial diversity and orchestrated structural changes during early life are key features for a healthy microbiome. Ethnicity and regional differences have a key impact on the vaginal microbiota. It remains to be determined whether changes in the vaginal microbiome observed in Hispanic and black women account for the increased rate of preterm delivery in these ethnic groups [14–16].

The fetus swallows huge amounts of amniotic fluid. This explains why its gut gets colonized with the intrauterine microflora already before birth. After birth, the infant gets rapidly colonized by maternal vaginal, gut, and skin microbiota. In the term infant, the mode of delivery either by vaginal birth or by caesarian section determines microbial diversity and whether the gut is primarily colonized by the maternal vaginal and fecal or the skin microbiota. These data are a first scientific indication that early events have a long-term health impact on microbiota structures [17–20]. In contrast to the situation at term, the decision of preterm delivery by caesarian section or vaginal birth does not have an impact on gut microbial diversity and longitudinal microbiota changes and microbiota display a pattern distinct from that at term. As an example of the disparities, preterm microbiota lack* Bacteroides* species, which display a delayed colonization pattern after caesarian section. Although microbiomes are principally able to adapt to that of the term infant within several weeks, the intrauterine and postnatal miscolonization poses a major threat to the health of the preterm infant [21–23]. The following chapters summarize the substantial advances in our understanding of microbiota structures and dynamics during pregnancy and after birth.

## 3. Pathologic Changes of the Microbiome Associated with Premature Labor and Preterm Delivery

The reasons for preterm labor comprise a multitude of different causes including maternal psychosocial distress, hormonal changes, uterine overdistension, cervical disease, vascular and maternal disorders, and breakdown of the maternal-fetal tolerance [24]. Among these, infection and inflammation are the main drivers of preterm labor and account for at least one half of preterm births [24–26]. Till today, histopathology is the gold standard to determine chorioamnionitis in contrast to the low sensitivity and specificity of clinical evaluation scores and laboratory parameters [27]. In severe chorioamnionitis, a typical pattern with an increase in bacterial abundance and reduced diversity with the dominance of bacteria has been observed, which is not seen in the physiologic situation [28]. The evolutionary attenuation of the maternal and fetal immune system enables the intrauterine growth of the fetus, but immune tolerance of the mother at the same time puts the fetus at risk for infection [29]. The diagnosis of a maternal infection is of crucial importance, as the fetus, exposed to microbiota in the amniotic cavity, experiences an immunologic adaptation. This new phenomenon is termed the so-called immunotolerance or immunoparalysis. This means that the previous or ongoing exposure to microbiota suppresses the necessary physiologic immune response and that an adequate increase in classical markers of inflammation and infection is not guaranteed [30, 31]. The placental microbiota structures display distinct patterns in term and preterm born infants, independent of the mode of delivery, and can be influenced by living conditions like excessive weight gain during pregnancy [32, 33]. Microbial species of ureaplasma and mycoplasma but also Aerococcaceae, Bifidobacteriaceae, and Fusobacteria predominate or are even exclusively present in the membranes of preterm delivered babies. Conversely, bacterial dysbiosis and inflammation in the fetal membranes can occur without preterm labor and without the typical clinical signs and seems not to put the fetus at risk for preterm delivery [2, 4, 34]. In the situation of preterm labor, a shift of microbiota with reduced microbial diversity takes place. Latest molecular techniques proved superior to detect microbial invasion and diversity compared to conventional culture techniques, which opened a new diagnostic window of opportunity [35]. The long-prevailing concept of bacterial ascension or transmission from the urogenital tract as the main driver of chorioamnionitis and amniotic inflammation was based on the bacteriological detection of microbes typically present. They include bacterial species of the genera* Streptococcus, E. coli, Gardnerella *spp.*, Prevotella, Gonorrhea, Treponema, Chlamydia, Ureaplasma, and Mycoplasma* as well as yeasts like* Candida* [13, 36–38]. Further species including anaerobic Fusobacteriaceae were recently detected by the non-cultivation-based techniques.

**Figure 1:**
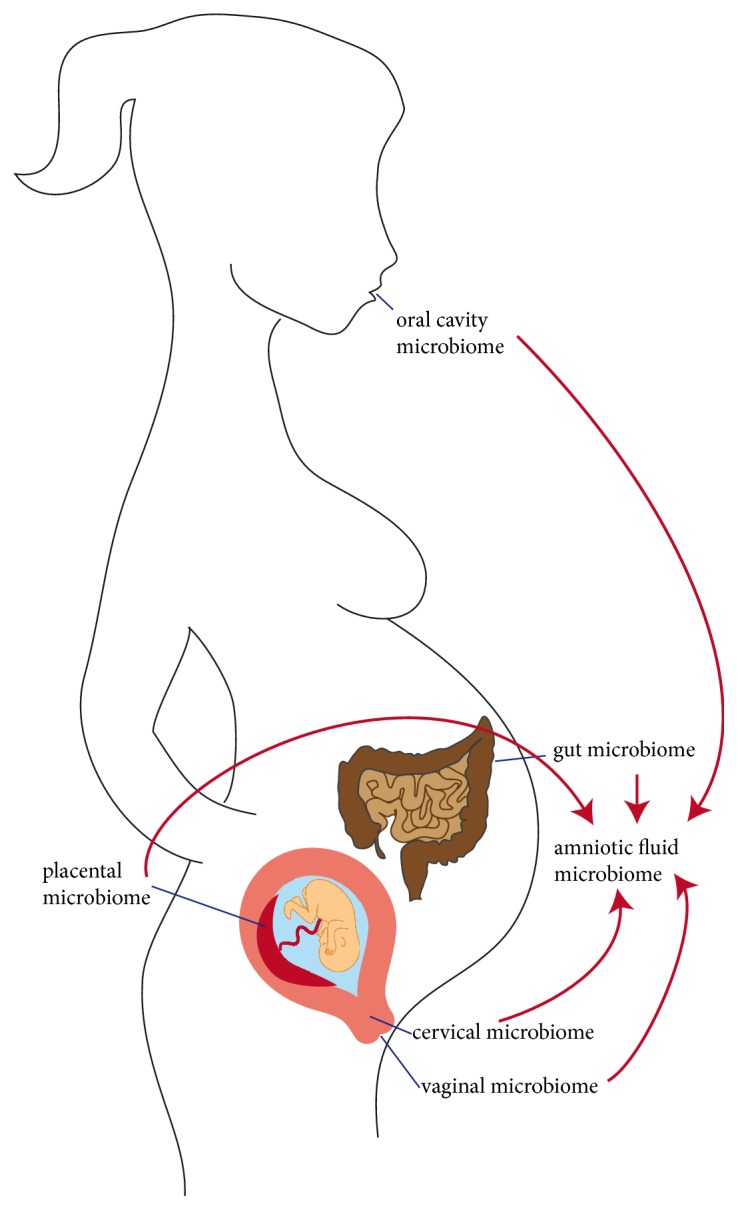
**Origin of microbiota in the amniotic cavity leading to preterm birth:** The microbiome in the amniotic cavity has long been thought to originate exclusively from the vaginal and cervical microbiome. But microbiota from the oral cavity, gut, and even the placenta provide a substantial contribution to the microbiome in the amniotic cavity mainly via haematogenic spread.

In fact, the microbiome gets dominated by abundance of bacteria from the urogenital tract, the gut, and the oral cavity (Figure 1). This shift is accompanied by alterations in microbial and metabolic pathways, which are suggested to contribute to preterm labor and birth [34, 39]. The lower urogenital tract and the perianal region constitute a microbial epitope which is highly influenced by the bacterial colonization of the gut and the local viability conditions. Microbiomes change during pregnancy, and differences in composition have long been acknowledged to account for variations in preterm birth rates [11, 16, 40, 41]. The reduction of microbial richness and diversity and changes in microbiome structure seem to occur early, in the first to second trimester of pregnancy, and there seem to exist racial disparities [14, 42–44]. That is, the cervical microbiota from women with* Chlamydia trachomatis* infection, which predisposes for preterm birth, differ completely from that of healthy women with respect to microbial diversity. They display a change in microbial taxa away from* Lactobacillus* species to anaerobes [43, 45]. While* Lactobacillus* species that dominated cervical microbiota have a lower risk of invasion of the amniotic cavity and chorioamnionitis after premature rupture of membranes, the prevalence of* Gardnerella *and* Sneathia* increases the probability [46].

Microbiota from the oral cavity can equally induce chorioamnionitis, when they are translocated via the bloodstream or by sexual practices. Members of the genera* Streptococcus, Porphyromonas, Filifactor, Campylobacter, *and* Fusobacterium* represent species which were repetitively connected with preterm labor. The scientific data point out that gut microbiota constitute the third source for bacterial translocation and can cause amniotic infection and chorioamnionitis. Reduction in microbial diversity and changes in gut microbiota occur during pregnancy with a dominance of Proteobacteria and Acinetobacteria away from* Bifidobacterium*,* Streptococcus*, Clostridiales, and* Bacteroides*, which predisposes for preterm delivery and disease [47–51]. The pathologic changes in the amniotic microbiome are retrieved in meconium samples from preterm infants with a high accordance. Microbiota are shifted to strains of the genera* Enterobacter*,* Enterococcus*,* Photorhabdus,* and* Tannerella*, which are known for their inflammatory, and potentially preterm birth inducing, properties [39, 52, 53]. In line with these observations, the type of nutritional diet or an active inflammatory bowel disease impacts on the gut microbiota composition, and the risk of preterm delivery is increased in these patients. This underlines the concept that a change in maternal gut microbiota is one of the responsible triggers [54].

## 4. The Consequences for the Premature Infant

Inflammatory diseases represent the biggest threat to the preterm infant and affect all organs, including the immature lung, cardiovascular system, immune system, brain, eye, and gastrointestinal tract, with acute and persistent consequences for the patient's health. Infection accounts for or aggravates acute respiratory distress, leakage, and arterial hypotension after birth. Simultaneously, all organs are at high risk for secondary complications including NEC, nosocomial infection, cerebral damage, retinopathy of prematurity, and endocrinological nonbalance. Infection promotes the establishment of BPD and somatic and developmental disorders [55].

**Figure 2:**
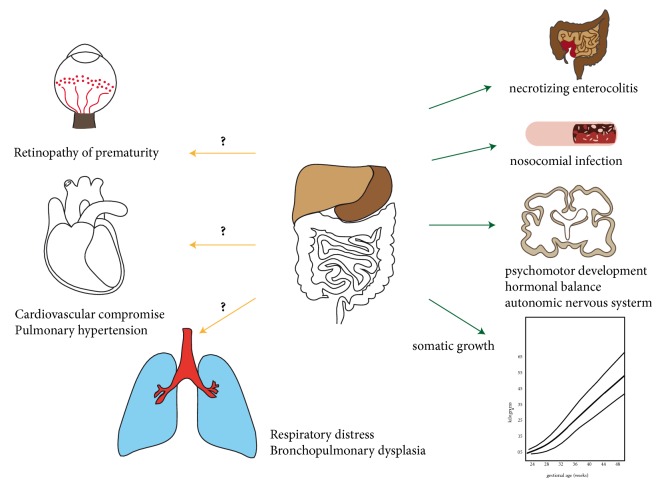
**Impact of gut microbiota on the acute and long-term morbidities in the preterm infant:** The gut microbiota exerts a central influence on human health. In the preterm infant, their impact on NEC and nosocomial infection is well recognized. First studies hint to an important impact on somatic growth, psychomotor development, autonomic regulation, and hormonal balance. In contrast, the contribution to the other acute and long-term sequelae remains to be determined.

Antibiotic therapy is aimed at combating life-threatening pre- or postnatal infections with pathogenic microbiota, but they cannot reduce or prevent the concomitant inflammatory organ damage. So far, most pathomechanistic insights are available for the inflammatory damage to the immature lung and efficient therapeutic interventions are restricted to a very limited number of drugs. The reason for the so far unsuccessful establishment of effective therapeutic interventions is based on the complexity of the involved pathomechanisms. The complex interplay between different central pathways and persistent cell phenotype distortion after a one-time injury poses further obstacles that need to be bypassed to reach therapeutic efficiency [56–59]. The following sections are dedicated to the detailed description of some of the most important disease burdens provoked by microbial dysbiosis. They summarize the actual status of therapeutic interventions with proven efficacy. Special focus is drawn to highlight the impact of the disturbed endogenous gut microbiome (Figure 2).

## 5. Microbiota of the Airways and Bronchopulmonary Dysplasia

Bronchopulmonary dysplasia (BPD) is the chronic lung disease of the preterm infant leading to life-long limitations in lung function [59]. On a pathophysiologic level, BPD is characterized by distorted alveolar and vascular growth in the saccular stage of lung development. Central to the pathogenesis is the pulmonary inflammatory response after birth, which is mainly provoked by pre- and postnatal infections and the life-saving therapies of mechanical ventilation and oxygen supply [58]. While chorioamnionitis and special pathogens like bacterial species derived from ureaplasma are well acknowledged to contribute to the disease in animal trials and preterm cohort studies, the impact of microbial colonization of the respiratory tract* in utero* and after birth was neglected until recently [60–63]. This is surprising, considering the tremendous impact of the microbiome on other pulmonary diseases and immunity of the lung including asthma, pneumonia, cystic fibrosis, COPD, and even pulmonary fibrosis. The airway of the preterm infant is not sterile at birth and its microbiome is highly influenced by the microbiome of the amniotic fluid. Differences in colonization and clinical parameters allow the categorization into disease clusters, which are predictive for the clinical course and outcome [64]. Reduced microbial diversity at birth, initial abundance of ureaplasma species in tracheal aspirates of ventilated preterm infants, and more pronounced changes in the longitudinal microbial community are associated with higher severity of BPD. The association of a predominance of Proteobacteria and decrease in* Lactobacillus* species in the airways of infants with severe BPD was recapitulated in the murine animal model with major impact on the regulation of central lung signaling pathways [65–68]. The well-accepted further dimension arising from the interaction of gut microbiota with the lung in other pulmonary diseases termed the gut-lung axis needs to be established for BPD.

## 6. The Significant Impact of Microbiota on Necrotizing Enterocolitis

In contrast to BPD, the important contribution of bacteria to the pathogenesis of necrotizing enterocolitis, which constitutes one of the most devastating morbidities of prematurity, is well established. NEC is considered to be a multifactorial disease. The inflammatory response of the gut to microorganisms is a central hypothesis of necrotizing enterocolitis pathogenesis [69, 70]. The great beneficial advantages of breast milk provision are attributed to microbiota diversity and the shaping of immunologic properties [71]. In contrast, antibiotic therapy drives microbial dysbiosis and increases the risk of necrotizing enterocolitis [72]. Derived from these findings, the benefits of prophylactic application of probiotic strains of Bifidobacteria, Lactobacillus, and Saccharomyces was tested in varying experimental and clinical settings. Despite the heterogeneity of results and the need for large-scale meta-analyses, the most recent reviews clearly established a benefit of probiotics to nearly halve the risk of necrotizing enterocolitis and to reduce the incidence of nosocomial infection and death [73, 74].

Postnatally, the gut gets colonized by Gram-positive and Gram-negative bacteria mostly with a facultative or strictly anaerobic metabolism. In the term infant the gut is dominated by Bifidobacteriales and* Bacteroides*. In the preterm infant the presence of a large number of different genera including* Anaerococcus, Aquabacterium, Bacillus, Bifidobacterium, Corynebacterium, Micrococcus, Oceanobacillus, Propionibacterium, Pseudomonas, Rothia, Sarcina, Sneathia, *and* Streptococcu*s has been described. As a general phenomenon, the gut microbiome of the preterm infant is dominated by Proteobacteria even when breast milk provision is assured and the appearance of* Clostridium* and* Vellonella *species is retarded [53, 75–78]. The microbiome is highly impacted by pre- and postnatal antibiotic therapy [79]. In this context, probiotic therapy aims to establish and maintain physiologic gut microbiota structures.

A strong dominance of Gram-negative bacteria and a decrease in anaerobic bacteria are described before onset of clinical symptoms of necrotizing enterocolitis, but whether altered microbial structures predispose for necrotizing enterocolitis or are a consequence of gastrointestinal or immunologic immaturity remains an open question. The discrepancies in microbiome structures between the feces and samples obtained from the oral cavity or stomach and the impact of microbial dysbiosis of the upper gastrointestinal tract are awaiting further clarification. Presence of Gram-negative bacteria and staphylococci allows the conclusion of their acquisition from the NICU environment [80]. Microbial colonization can be separated into peripartal acquisition as described, i.e., for* Escherichia coli* and* Candida albicans* and hospital acquired microbial structures including* Klebsiella*,* Enterobacter,* and* Acinetobacter* species and* Candida *species other than* Candida albicans* [81]. Taken together, studies of the intestinal microbiome revealed a reduction of bacterial diversity and a shift of microbiota from Bacteroidetes and Firmicutes towards Proteobacteria and potentially pathogenic species including* Staphylococcus aureus, Escherichia coli, Shigella *spp.*, Citrobacter* spp., and* Klebsiella* spp. before the onset of clinical symptoms of necrotizing enterocolitis [78, 82–85].

## 7. Microbial Dysbiosis and the Risk for Nosocomial Infection

Nosocomial infections pose a special risk to the premature infant. Due to the immaturity and immunologic incompetence of the immune system, the preterm infant is particularly vulnerable to nosocomial infections in the hostile environment of the neonatal intensive care unit [30, 31, 86–90]. Furthermore, therapies with antenatal steroids and magnesium as well as small-for-gestational-age status and antenatal smoke exposure seem to further impact and diminish the immunologic response capacity [91–94]. Therefore, skin and gut microbiota colonization and maturation are important prerequisites to prevent pathogen overgrowth and nosocomial infection. The skin of the healthy term and preterm infant is dominated by Firmicutes, Actinobacteria, Proteobacteria, and Bacteroidetes exposing them to nosocomial infection [95, 96]. The physiologic dominance of gut microbiota structures by Bifidobacteria may serve as a protective factor from gut-epithelial translocation. The differences in gut microbiota between preterm and term born infants at the onset of sepsis are used as an explanation for the vulnerability of the preterm infant and the important role of the gut microbiome in disease initiation. Concordance of microbiota isolated from the gut of infants with sepsis and bacteria identified in positive blood cultures supports this assumption [97–99]. The actual pathomechanistic understanding suggests the following sequence: The preterm infant is exposed to the hostile environment of the neonatal intensive care unit and gut microbiota display a big disparity after birth. A uniform microbiome is established within the first weeks of life with prevalence of highly pathogenic bacteria including* Staphylococcus, Enterococcus,* and several Enterobacteriaceae, while Bifidobacteria are infrequently detected. Reduction in microbial diversity with predominance of Staphylococci is again another feature predisposing for late-onset infection [100]. Not surprisingly, pre- or postnatal antibiotic therapy reduces microbial diversity and impedes the establishment of physiologic gut microbiota with a shift from Firmicutes and Bacteroidetes towards Proteobacteria and Actinobacteria [85, 101–103].

**Figure 3:**
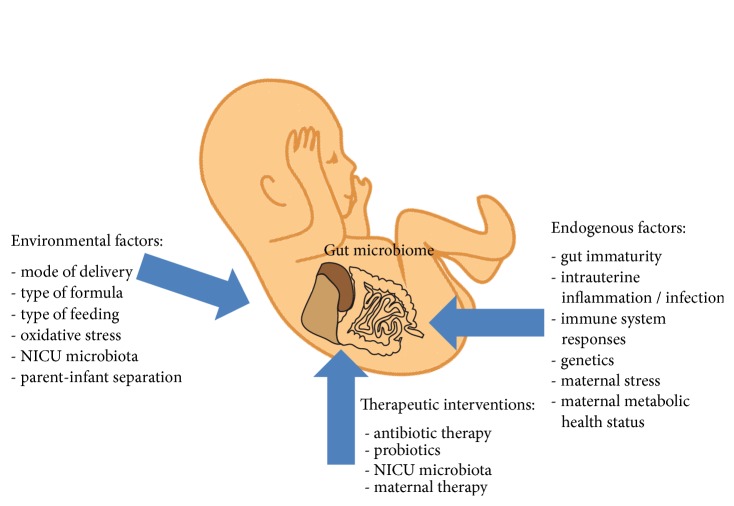
**Factors determining the composition of gut microbiota in the preterm infant:** The microbiome of the gastrointestinal tract of the preterm infant varies widely from that at term and is impacted by a plenty of endogenous and environmental factors and pre- and postnatal therapeutic interventions.

Taken together, the gut microbiome of the preterm infant is highly impacted by endogenous and environmental factors and maternal and postnatal therapeutic interventions which accounts for the high susceptibility for nosocomial infection (Figure 3).

## 8. The Gut-Brain Axis in Prematurity

Brain development and function undergo fundamental steps in the last trimester and in the first year of life. The physiologic steps of brain folding and the developmental steps of the brain connectome far outreach the increase in brain volume and highly impact functionality. Their functional importance can be derived from preterm infants with severe limitations in brain functions, which display tremendous alterations in these critical steps [104, 105]. More and more data from animal and human studies support the finding that the gut microbiome, especially at early postnatal stages, has tremendous impact on behavioral and stress responses later in life [106–110]. The term gut-brain axis summarizes not only the multiple and complex functions of the cerebrum, but endocrine homeostasis, the sympathetic-parasympathetic, and even the enteric nervous system. The neurologic disorders comprise diseases like autism spectrum disorders, depression, and anxiety which are frequently observed in former preterm infants and restrict their quality of life beyond intelligence and gross and fine motor functions [111, 112]. Even persisting hormonal dysregulations in former preterms are coming into the focus of research [113]. Underlining the functional relevance of the gut-brain axis to the neurodevelopmental outcome after prematurity, higher and persisting prevalence of* Bifidobacteria* in the gut microbiome is associated with improved scores for mental development at 24 months [114]. Convincing animal data demonstrate the far-reaching impact of pre- and postnatal microbiota changes on brain development and the different functional regions which were reproduced in first association studies in children [115–117]. The gut-brain axis is not a one-way but the gut and the brain impact each other bidirectionally which can lead to multiplication in effect size [118–120]. This comes especially true as the preterm infant is exposed to high stress levels and repeated painful procedures [121]. Future studies will have to elucidate how microbiota modulate brain development and function physiologically compared to preterms, how these early life events lead to persisting psychomotor sequelae, and whether microglia cells are the only targets within the central nervous system [122].* Vice versa*, it remains to be determined how impaired brain function impacts physiologic microbiota structures.

## 9. Breast Milk and Beyond to Shape Physiologic Microbiota Structures

Breast milk is the optimal nutrition of the preterm infant with respect to acute and long-term health, somatic growth, and psychomotor development. It is well established from studies in healthy newborn that the infant's microbiome is crucially promoted and shaped by the microbiota, anti-inflammatory and antioxidative properties, growth factors, hormonally active substances, and cytokines provided by breast milk feeding. Overall, physiologic gut microbiota establishment and enrichment are facilitated by breast milk [69]. The maternal microbes excreted from the mammary gland, the contact to the skin of the breast, and the nutritional components of breast milk enable the maturation of immune functions and the establishment of a stable physiologic rich and diverse microbiome with a dominance of* Bifidobacteria* and* Lactobacillu*s species but also presence of species of the genera* Staphylococcus, Streptococcus, Propionibacterium, Bacteroides, Rothia, Enterococcusi, *and* Pseudomonas* [99, 123–129]. But also strictly anaerobic gut commensals from the Clostridiaceae including* Blautia, Clostridium, Collinsella* and* Veillonella* species were detected in breast milk together with* Coprococcus, Faecalibacterium,* and* Roseburia* species, which were simultaneously isolated from the mothers' breast milk and stool. This allows the conclusion that additionally to the skin-gut axis a maternal gut-breast microbiome axis exists and that the infants' gut microbiome is shaped by the maternal gut microbiome [128, 130, 131]. Gut microbiota composition after preterm birth differs from that of mothers who delivered at term with a shift from* Bifidobacterium* to* Enterococcus *species. It is impacted by perinatal maternal antibiotic therapy with a decrease in* Lactobacillus, Bifidobacterium, Staphylococcus,* and* Eubacterium *species [132]. Formula fed infants display a further reduced microbial diversity and the dominance of Enterobacteriaceae, Coriobacteriaceae, and* Bacteroides* [95, 126]. In preterm infants at high risk of gut microbial dysbiosis, probiotic therapy with the bacterial commensals identified in the previously mentioned studies proved overall efficient to reduce the incidence of necrotizing enterocolitis, sepsis, and death. However, the optimal strain or formula and the duration of application await further exploration [73, 74]. Lactoferrin, a protein of the transferrin family with broad antimicrobial action, stands for the steep rising gain of knowledge about the gut microbiota shaping functions of breast milk. Its recombinant application does not only reduce the risk for device associated infections but modulates the fecal microbiome towards the physiologic situation [133]. In contrast to these medical therapeutic interventions, skin-to-skin care is an easy to apply clinical technique to shape the infants' microbiome. Its consistent provision shapes the oral microbiome of the preterm infant and helps to accelerate its maturation [134].

## 10. Concluding Remarks

The available data convincingly support the hypothesis that the pre- and postnatal microbiome contributes to premature delivery and to the acute sequelae in the preterm infant. Despite the tremendous scientific progress, we have just scratched the surface to understand the consequences of aberrant microbiota and their dynamic changes to disease initiation and progression. The current data convincingly highlight their impact on nosocomial infection and NEC, which constitute not only a tremendous disease burden to the preterm infant but more importantly entail life-long consequences and considerable lethality. Nonetheless, for most of the acute complications and short-term sequelae including pulmonary and cerebral problems a clear cause-relationship is still missing. It remains to be determined whether pathogenic microbiota also account for the distortion of long-term somatic and psychomotor development in preterm infants which did not suffer from severe acute complications like infection or cerebral hemorrhage. Therefore, comprehensive and long-term oriented research efforts are urgently needed to cover these important and clinically highly relevant aspects. A comprehensive and mechanistic understanding of the connection between microbial dysbiosis and disease initiation and progression will help to develop new therapeutic concepts aiming to control and restore physiologic microbial structures. Further important topics of research are to gain detailed knowledge on microbial structures and how to avoid of sample contamination and to enable the comparability of studies with respect to techniques and sample preparation [135]. It remains an open question how our lifestyle habits and the genetic background impact the microbiome of the pregnant woman and the frequency of preterm born infants.

The successful implementation of postnatal probiotic therapy and further clinical guidelines raises hopes to reduce the maternal and fetal disease burden in the near future and to come to a targeted or even personalized medicine. Next steps can be derived from the observational studies and should include (1) the design of point-of-care techniques to determine microbial structures onsite and in real time to immediately identify the mother and infant at risk, (2) evaluation of the benefits of personalized medicine strategies of vaginal fluid or feces transplantation to the fetus and newborn, (3) the development of new strategies to detect bacterial infection with high prediction accuracy to avoid unnecessary and prolonged antibiotic therapy and subsequent microbial dysbiosis, and (4) to test more potent alternatives to current classical probiotics including mixtures of different bacterial strains and bacterial metabolites. Each of these areas poses tremendous challenges and opportunities to finally reduce the rates of prematurity and of the associated morbidities. Opening and reaching these new frontiers in perinatal science offer the opportunity to come closer to efficient prevention of preterm birth, which poses an ever greater global burden and challenge.
